# Metabolic Profiling of Hybrids Generated from Pummelo and *Citrus latipes* in Relation to Their Attraction to *Diaphorina citri*, the Vector of Huanglongbing

**DOI:** 10.3390/metabo10120477

**Published:** 2020-11-24

**Authors:** Nabil Killiny, Shelley E. Jones, Faraj Hijaz, Abdelaziz Kishk, Yulica Santos-Ortega, Yasser Nehela, Ahmad A. Omar, Qibin Yu, Fred G. Gmitter, Jude W. Grosser, Manjul Dutt

**Affiliations:** 1 Department of Plant Pathology, University of Florida, Citrus Research and Education Center, IFAS, Lake Alfred, FL 33850, USA; shjones@ufl.edu (S.E.J.); fhijaz@ufl.edu (F.H.); kishk_a_2013@hotmail.com (A.K.); yulica.santosort@ufl.edu (Y.S.-O.); yasser.nehela@ufl.edu (Y.N.); 2 Department of Plant Protection, Faculty of Agriculture, Tanta University, Tanta 31511, Egypt; 3 Department of Agricultural Botany, Faculty of Agriculture, Tanta University, Tanta 31511, Egypt; 4 Department of Horticultural Sciences, University of Florida, Citrus Research and Education Center, IFAS, Lake Alfred, FL 33850, USA; omar71@ufl.edu (A.A.O.); qibin@ufl.edu (Q.Y.); fgmitter@ufl.edu (F.G.G.J.); jgrosser@ufl.edu (J.W.G.); manjul@ufl.edu (M.D.); 5 Biochemistry Department, College of Agriculture, Zagazig University, Zagazig 44519, Egypt

**Keywords:** *Citrus latipes*, Pummelo, Asian citrus psyllids, citrus breeding, volatile organic compounds

## Abstract

The citrus industry at present is severely affected by huanglongbing disease (HLB). HLB is caused by the supposed bacterial pathogen “*Candidatus* Liberibacter asiaticus” and is transmitted by the insect vector, the Asian citrus psyllid, *Diaphorina citri* Kuwayama. Developing new citrus hybrids to improve HLB management is much needed. In this study, we investigated the metabolomic profiles of three new hybrids produced from the cross of C2-5-12 Pummelo (*Citrus maxima* (L.) Osbeck) × pollen from *Citrus latipes*. The hybrids were selected based on leaf morphology and seedling vigor. The selected hybrids exhibited compact and upright tree architecture as seen in *C. latipes*. Hybrids were verified by simple sequence repeat markers, and were subjected to metabolomic analysis using gas chromatography-mass spectrometry. The volatile organic compounds (VOCs) and polar metabolites profiling also showed that the new hybrids were different from their parents. Interestingly, the levels of stored VOCs in hybrid II were higher than those observed in its parents and other hybrids. The level of most VOCs released by hybrid II was also higher than that released from its parents. Additionally, the preference assay showed that hybrid II was more attractive to *D. citri* than its parents and other hybrids. The leaf morphology, compact and upright architecture of hybrid II, and its attraction to *D. citri* suggest that it could be used as a windbreak and trap tree for *D. citri* (double duty), once its tolerance to HLB disease is confirmed. Our results showed that metabolomic analysis could be successfully used to understand the biochemical mechanisms controlling the interaction of *D. citri* with its host plants.

## 1. Introduction

The huanglongbing (HLB, also known as citrus greening) is currently considered the most destructive disease to the citrus industry and is present in most of the citrus-growing areas of the world [1]. In North America, HLB is associated with the bacterial phytopathogen, “*Candidatus* Liberibacter asiaticus”, and transmitted by the Asian citrus psyllid, *Diaphorina citri* Kuwayama. The symptoms of HLB include blotchy mottle of leaves, yellow shoots, off-season bloom, stunting, misshapen and bitter fruit, and fruit drop [1]. Since the initial discovery of *D. citri* in Florida in June 1998, the infection rate of HLB in citrus groves has significantly increased up to 100% in some parts of the state, and has significantly reduced the citrus production in infected areas [1,2]. Currently, no cure exists for HLB and its management mainly depends on the control of its insect vector using insecticides. Unfortunately, the heavy use of insecticides for the control of *D. citri* can cause several critical problems such as insecticide resistance, residues in food products and environmental pollution [1]. Thus, effective and environment-friendly pest management strategies would reduce the cost of citrus production and minimize the use of insecticide. 

Most of citrus cultivars including sweet oranges, grapefruits and Pummelo (*C. maxima*) are susceptible to “*Ca.* L. asiaticus”, however, field observations indicated that some citrus varieties are tolerant to “*Ca.* L. asiaticus” bacterium [2]. Besides, controlled greenhouse studies confirmed that some citrus varieties such as *Citrus latipes* and Eureka lemon (*C. limonia* Osbeck) were more tolerant to the “*Ca.* L. asiaticus” pathogen than others [3]. In the same manner, field and greenhouse observations suggested that *D. citri* has specific host plant preferences [2,4]. For instance, field observations indicated that *Murraya paniculata* (L.) Jack (orange jasmine) and sweet orange were more preferred hosts by *D. citri* than *Poncirus trifoliata* [2]. Additionally, no-choice assays demonstrated that *P. trifoliata* and its hybrids were tolerant to *D. citri* [4]. The authors suggested that *P. trifoliata* have both antixenosis and antibiosis resistance to *D. citri* [4]. Furthermore, Tsai and Liu showed that grapefruit was a more preferred host for *D. citri* than sour orange, rough lemon and orange jasmine [5].

The life cycle of *D. citri* is related to the growth pattern of its host plants, and psyllid mainly mates, lays eggs and develops on fresh young shoots [6,7]. It is also known that *D. citri* is attracted to bright yellow and green colors, which are indicative of young shoots [8]. In terms of olfactory cues, previous studies suggested that volatile organic compounds (VOCs) emitted by flushing shoots could play an important role in the attraction of *D. citri* to its host plant [8]. In our previous study, we found that juvenile leaves produced more sesquiterpenes than mature leaves, whereas mature leaves produce more monoterpenes than juvenile leaves [9]. These results indicated that the ratio of monoterpenes/sesquiterpenes could be an important factor in the attraction of *D. citri* to its host plants. To understand why some citrus varieties are more tolerant to “*Ca.* L. asiaticus” than others, we investigated the volatile and non-volatile profiles of several citrus cultivars with different degrees of tolerance to “*Ca.* L. asiaticus” pathogen [10,11,12,13,14].

While the spread of HLB has been destructive to the citrus industry, it encouraged the citrus breeding programs to screen the entire field germplasm collection to identify selections with tolerance to this disease. Some tolerant selections have a direct cultivar potential, but other selections should be hybridized with complementary parents to achieve commercialization. Additionally, other selections could serve as new rootstock, trap plants or windbreaks. Recently, several new hybrids have been developed and released in Florida including “Sugar Belle”, “Bingo” and “411”. Fortunately, some of the newly released hybrids like “Sugar Belle” showed good tolerance to HLB disease in the field [13].

Advances in chromatography and mass spectrometry allow the detection and quantification of many polar and semipolar metabolites. Metabolomics has been widely used in different animal and plant species, and it is believed that there are about one million metabolites in the plant kingdom [15]. Recently, a new research area using metabolomics was initiated, ecometabolomics. This field focuses on the use of metabolomics in ecology to understand species’ interaction with the environment and with other organisms [16]. In this study, we investigated the metabolomic profiles of three new hybrids and their parents using GC-MS to explain the attraction of *D. citri* to those plants. The new hybrids were produced from the cross of C2-5-12 Pummelo (*C. maxima* (L.) Osbeck) × pollen from *C. latipes*. *C. latipes* has a compact architecture and is known for its tolerance to HLB [17], whereas Pummelo (*C. maxima*) generally makes large trees and is sensitive to HLB and is considered a good host for *D. citri* [2]. Crossing between Pummelo and *C. latipes* could result in new hybrids that are tolerant to “*Ca*. L. asiaticus” and/or its vector, which could be useful in the management of the HLB disease as citrus windbreak and trap tree.

## 2. Results 

### 2.1. Morphology of New Hybrids

**Figure 1:**
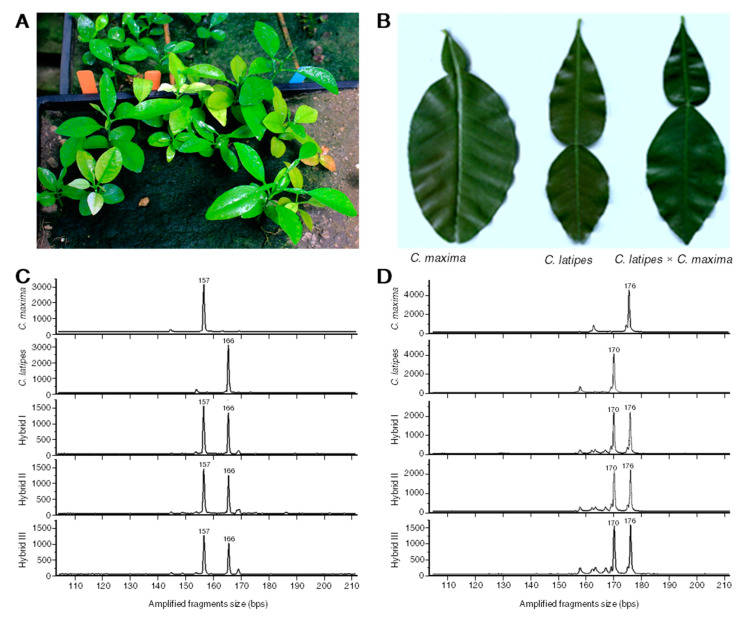
Selection and confirmation of new hybrids. (**A**) Selection of new hybrids based on their growth in calcareous soil (pH 8) that was inoculated with *Phytophthora nicotianae* and *P. palmivora* cultures. (**B**) Leaf morphology of the new hybrids evaluated in this study and their parents, *Citrus latipes* and *C. maxima.* (**C**,**D**) Electropherograms example of expressed sequence tag-simple sequence repeat (EST-SSR) using two SSR primers (loci; CX6F06Y and CX6F10R, respectively) of *Citrus latipes* and *C. maxima* and their corresponding sexual hybrids (Hybrid I, Hybrid II and Hybrid III). Numbers above the peak indicate the size of the amplified fragments measured in base pairs (bps).

Several putative “C2-5-12” Pummelo (*C. maxima*) × *C. latipes* hybrids were selected from a large population based on their growth in calcareous *Phytophthora*-infested soil (Figure 1A). Subsequently, leaf morphology (Figure 1B) and seedling vigor were used to narrow our selection to the three hybrids (I, II and III) utilized in this study. The morphology of the leaves of the new hybrids indicated a successful crossing between Pummelo (*C. maxima*) × *C. latipes* (Figure 1B). The morphology of the leaves of the new hybrids was intermediate between that of their parents (Figure 1B). The leaf of the new hybrids showed a medium wing, whereas the Pummelo parent had a larger blade and a small wing, and the *C. latipes* had a large wing (Figure 1B). The new hybrid showed vigorous growth and exhibited compact and upright tree architecture.

### 2.2. Simple Sequence Repeat 

Eight primer sets (CX6F02B, CX6F04G, CX6F06Y, CX6F10R, CX6F17B, CX6F18G, CX6F29Y and CX0035R) revealed alleles in the hybrids that matched those of the parents. This result indicated that the offspring were related to their parents (Table 1). Three of these primers (CX6F02B, CX6F29Y and CX0035R) revealed no polymorphism and were uninformative. 

Four of the primers (CX6F06Y, CX6F10R, CX6F17B and CX6F18G) produced allele combinations in all three progeny that would only be possible in a hybrid. The first two of these primers definitively support crossbreeding by heterozygosity derived from two homozygous parental plants (Figure 1C,D). The third primer supports crossbreeding by virtue of the pollen parent unique allele 126 being present in each seedling. The fourth primer supports crossbreeding by heterozygosity arising from a homozygous pollen source’s allele present in the three hybrids. Homozygosity of the seedlings for CX6F04G allele 169 potentially could have resulted if the seedlings arose from self-pollination of the seed parent. However, given the evidence at the other four loci, results at this locus also are supportive of the crossbreeding. In summary, the results of SSR marker analysis confirmed that the seedlings were hybrids of C2-5-12 *C. maxima* × *C. latipes*.

### 2.3. Polar Metabolites

Polar metabolites detected in *C. latipes*, Pummelo, and their hybrids are shown in Table S1. The levels of lactic acid, phosphoric acid, _L_-proline, _L_-aspartic acid, citric acid, saccharic acid, galactaric acid and sucrose were highest in *C. latipes* (Table S1). On the other hand, the levels of _L_-threonine, malic acid, threonic acid, ribonic acid, shikimic acid, quinic acid, gluconic acid and *scyllo*-inositol were highest in Pummelo (Table S1). The level of sucrose in *C. latipes* and new hybrids was significantly higher than that in Pummelo (Table S1). The highest level of *chiro*-inositol was found in hybrid II and *C. latipes* (Table S1).

**Figure 2:**
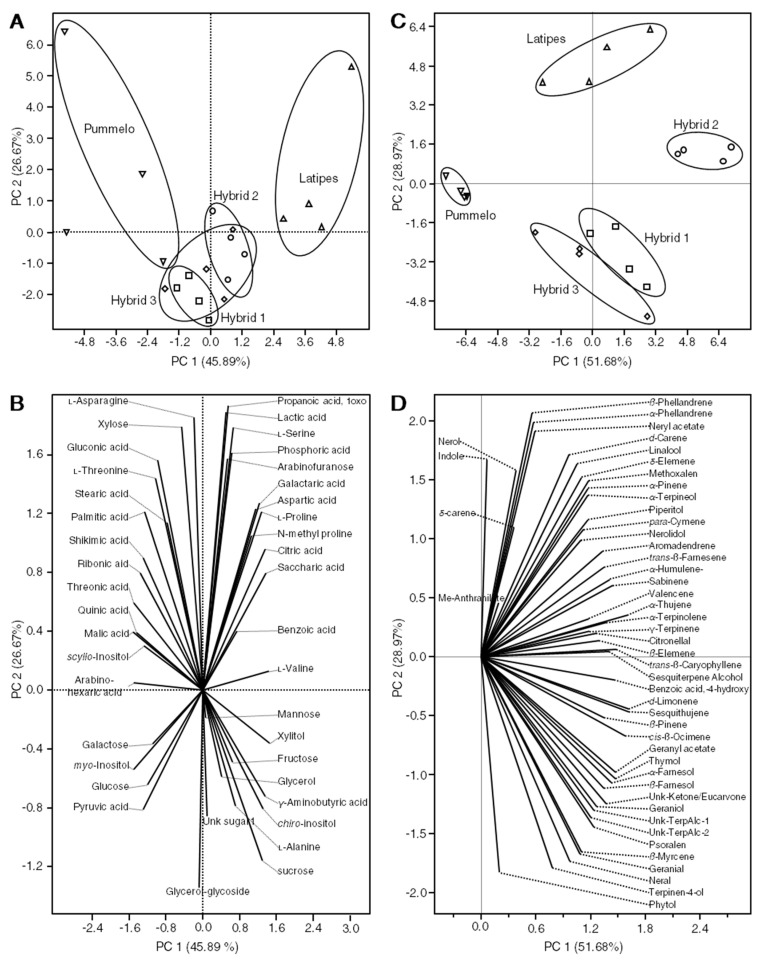
Principal components analysis and its associated loading plot for polar and volatile metabolites in three different hybrids and their parents using GC-MS (*n* = 4). (**A**) Principal components analysis and its associated loading plot (**B**), using the concentration of all detected leaf non-volatile metabolites. (**C**) Principal components analysis and its associated loading plot (**D**) using the concentration of all detected leaf volatile (hexane extract) metabolites. In panels (**A**,**C**), upward-facing triangles represent *C. latipes*, downward-facing triangles represent *C. maxima* (Pummelo), squares represent hybrid 1, circles signify hybrid 2, and rhombuses represent hybrid 3.

The score plot also showed that the polar metabolite profile of *C. latipes* and Pummelo were different from each other (Figure 2A). On the other hand, the new hybrids clustered close to each other, indicating that they had similar polar metabolite profiles (Figure 2A). The new hybrids clustered in between their parents, suggesting a successful crossbreeding between Pummelo and *C. latipes*. (Figure 2A). Principal component 1 and 2 accounted for 74.6% of the total variance (Figure 2A). The loading plot (Figure 2B) showed that Pummelo was high in several metabolites such as quinic and shikimic acid (left top quadrant), whereas *C. latipes* was high in metabolites that clustered at the top right quadrant such as proline and citric acid (Figure 2B). Hybrids were high in metabolites that clustered at the bottom of the loading plot such as glucose, sucrose and alanine (Figure 2B).

### 2.4. Volatile Content of Hexane Extract

The GC-MS analysis revealed a total of 47 volatile compounds in the hexane extract of citrus leaves from the two parents and three hybrids and included monoterpenes (peaks 1–13), terpene alcohols and aldehydes (pks 14–27), esters such as neryl acetate and geranyl acetate (pks 29 and 30), sesquiterpenes (pks 31–39) and sesquiterpenes alcohols (pks 41–47) as listed in Table 2. Leaves of *C. latipes* contained all 47 VOCs while Pummelo contained the fewest number (28) of VOCs. The hybrids I, II and III contained 43, 45 and 42 VOCs, respectively (Table 2). The total levels of the volatile compounds in the new hybrids were significantly higher than their parents (Table 2).

**Figure 3:**
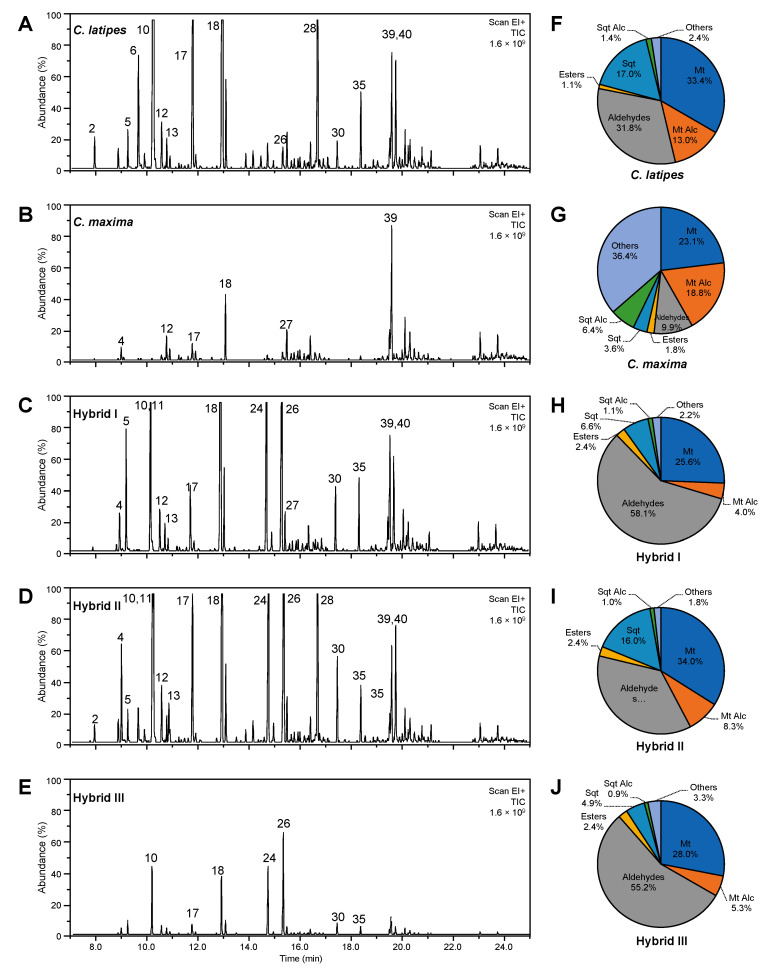
Volatile organic compounds (VOCs) detected in *Citrus maxima*, *C.itrus latipes* and their corresponding sexual hybrids (Hybrid I, Hybrid II and Hybrid III). (**A**–**E**) GC-MS representative chromatograms of the volatile organic compounds detected in the hexane extracts of leaves collected from *C. latipes* and *C. maxima* and their hybrids (Hybrid I, Hybrid II and Hybrid III), respectively. (**F**–**J**) Percentages of different volatile groups detected in the hexane extracts using GC-MS of leaves collected from *C. latipes* and *C. maxima* and their hybrids (Hybrid I, Hybrid II and Hybrid III), respectively. Abbreviations: Mt, Monoterpenes; Mt Alc, Monoterpene alcohols; Sqt, Sesquiterpenes and Sqt Alc, Sesquiterpene alcohols.

The score plot also showed that the volatile metabolite profile of *C. latipes* and Pummelo were different from each other and were also different from the new hybrids (Figure 2C). Hybrid I and III clustered close to each other, whereas hybrid II clustered alone at the right bottom of the first quadrant (Figure 2C). Principal component 1 and 2 accounted for 72.6% of the total variance (Figure 2C). The loading plot (Figure 2D) showed that *C. latipes* and the new hybrids contained a higher level of volatiles than Pummelo (Figure 2D). As shown in (Table 2 and Figure 3A,B), Pummelo was low in most of the detected volatiles, whereas *C. latipes* was high in several volatile compounds including *α*- and *β*-phellandrene, *d*-limonene, linalool, citronellal and δ-elemene. 

In addition, the GC-MS analysis of leaf extracts showed that the volatile profiles of the parents were also different from their hybrid offspring (Table 2 and Figure 3A–E). In general, the new hybrids contained high amounts of VOCs and were more similar to *C. latipes* than Pummelo since they contained the majority of the detected compound (Table 2 and Figure 3C–E). Furthermore, the volatile profile of hybrid II was different from those of the other hybrids. In general, hybrid II contained higher amounts of volatiles compared with hybrids I and III (Table 2 and Figure 3D). For example, hybrid II had more α- and β-pinene, γ-terpinine and δ-elemene compared to both parental citrus and the other hybrids (Table 2). The volatile profile of hybrid II was closest to the *C. latipes* parent VOC profile (Table 2 and Figure 3A,B,D). Interestingly, several of the compounds including *γ*-Carene, *p*-cymene, β-phellandrene, citronellal, δ-elemene, valencene, methyl anthranilate, nerol and geraniol were present. This indicates that they were inherited from *C. latipes* (Table 2 and Figure 3). 

The percentages of volatile groups in the new hybrids and their parents are shown in (Figure 3F–J). The highest percentage of monoterpenes was found in hybrid II (34.0%) and *C. latipes* (33.4%) (Figure 3F,I). The highest percentage of sesquiterpenes was also present in *C. latipes* (17.0%), followed by hybrid II (16.0%) and hybrid I (6.6%; Figure 3F,H,I). The highest percentage of aldehydes was found in hybrid I (58.1%), followed by hybrid III (55.2%), hybrid II (36.5%), *C. latipes* (31.8%) and Pummelo (9.9%; Figure 3F–J). 

### 2.5. Released Volatiles Collected by Static Headspace SPME

**Figure 4:**
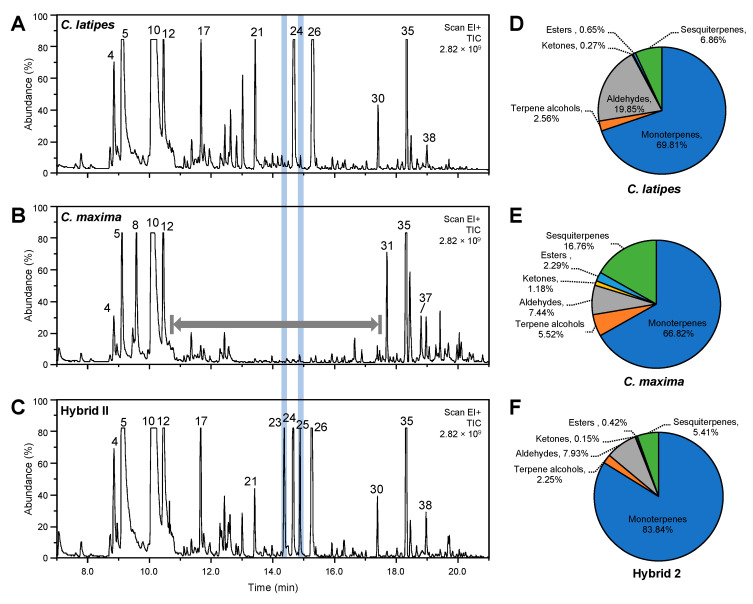
Released volatile organic compounds detected via solid phase microextraction (SPME) in *Citrus maxima*, *C. latipes* and their promising sexual hybrid (Hybrid II). (**A**–**C**) GC-MS representative chromatograms of the volatile organic compounds released from the leaves of *C. latipes* and *C. maxima* and their promising hybrid (Hybrid II). (**D**–**F**) Percentages of different volatile groups released from *C. maxima, C. latipes* and their promising hybrid (Hybrid II). The peak numbers on the representative SPME chromatograms correspond to Table 2. The vertical blue bars refer to peak 23 and peak 25. The gray horizontal bar in *C. latipes* chromatogram (**C**) indicates the interval (11–18 min) where most of the differences in released volatiles were observed.

In this section, we only focused on the volatile organic compounds released from *C. maxima*, *Citrus latipes* and their promising hybrid, hybrid II, which showed the highest content of monoterpenes (insect attractants). A total of 41 volatile compounds were detected by headspace solid phase microextraction (SPME), 14 of which were not detected in hexane extracts of the leaves. Interestingly, nine of the 14 compounds detected only by in vivo SPME were sesquiterpenes. The overall volatile profiles of the two parents and hybrid II collected by SPME were similar to the profiles obtained from the hexane extracts. The levels of most released volatiles from the Pummelo parent were lower than those released from *C. latipes* or hybrid II. Figure 4 shows a typical SPME-GC-MS chromatogram of the two parents and hybrid II (Figure 4A–C) along with the corresponding total relative peak area percentages of the major VOC classes (Figure 4D–F). Figure 4 shows that our SPME method was sensitive to a broad range of VOCs, except sesquiterpenes alcohols (peaks 41–47 in Table 2), which were not extracted by the SPME method and were only found in the hexane extracts.

Leaves released similar amounts of *d*-limonene (peak 10), (Z)-*β*-ocimene (peak 12) and trans-*β*-caryophyllene (peak 35). Figure 4A shows that *C. latipes* leaves released more terpinen-4-ol and *α*-terpineol (pks 20–21) and was the source of the geranyl acetate (pk 30; Figure 4A). On the other hand, the levels of released *δ*-carene (peak 8), *β*-elemene (peak 31) and trans-*β*-farnesene (peak 37) were higher in Pummelo than in *C. latipes* or hybrid II, while alcohols such as linalool and α-terpineol were lower (peaks 17 and 21), or absent (peaks 23 and 25), and the aldehydes neral and geranial were very low (peaks 24 and 26; Figure 4B). Likewise, the level of several released volatiles such as *β*-pinene (peak 4), *β*-myrcene (peak 5), nerol and geraniol (peaks 23 and 25 and blue bars) in hybrid II were higher than those released from its parents (Figure 4C). As percentages, total monoterpenes in hybrid II (83.8%) were higher than in both *C. latipes* (69.8%) and Pummelo (66.8%) (Figure 4D–F). The percentage of total sesquiterpenes in Pummelo (16.8%) was higher than that in *C. latipes* (6.8%) and hybrid II (5.4%; Figure 4D–F). The percentage of total aldehydes in *C. latipes* (19.9%) was higher than hybrid II (7.9%) and Pummelo (7.4%; Figure 4D–F).

**Figure 5:**
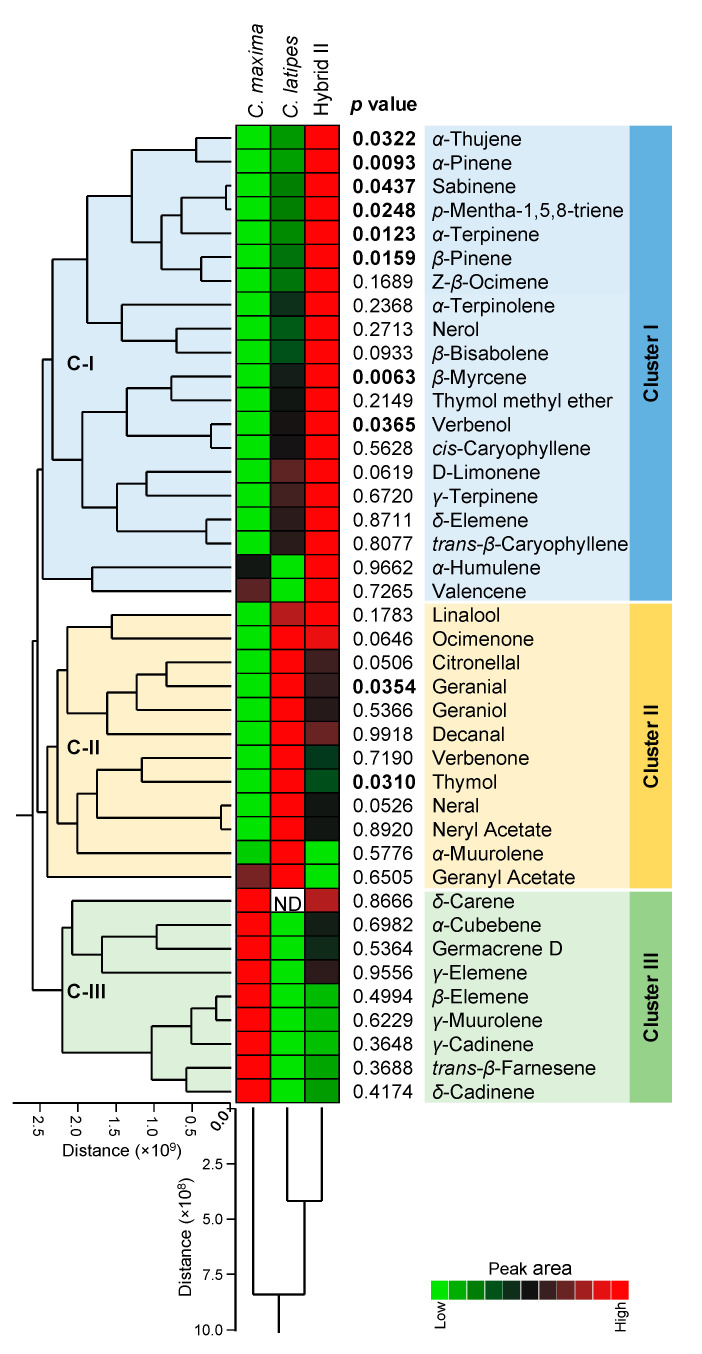
Two-way hierarchical cluster analysis (HCA) and heat map of individual volatile organic compounds released from *C. maxima, C. latipes* and their promising sexual hybrid (Hybrid II). The differences in the abundances of volatile organic compounds between the three treatments are visualized in the heat map diagram. Rows represent the individual compounds, while columns represent the cultivars and the hybrid. Peak areas were obtained by in vivo SPME-GC-MS. Higher peak areas are colored red and lower peak areas are colored green (see the scale at the right corner of the bottom of the heat map). Volatile organic compounds and treatments were organized using HCA based on Ward’s minimum variance method. The *p*-values are listed to the right side of the heat map. The bolded *p*-values indicate statistically significant differences among treatments, while the normal (non-bolded) *p*-values signify no significant differences among treatments using Tukey–Kramer honest significant difference test (Tukey HSD; *p* < 0.05), except for the *δ*-carene, which was analyzed using the two-way *t*-test (*p* < 0.05).

The heat map (Figure 5) was generated using the peak areas obtained by in vivo SPME. Figure 5 shows that cluster I was dominated by VOCs released in higher amounts by hybrid II, which included *β*-myrcene, *α*-pinene, *α*-terpinene and *β*-pinene among others. A majority of the compounds in Cluster I were monoterpenes. Cluster II of the heat map revealed the compounds that were higher in *C. latipes*, including geranial and thymol. The majority of these were terpene alcohols and aldehydes. Cluster III shows the nine VOCs that were higher in Pummelo as compared to *C. latipes* and hybrid II, of which eight were sesquiterpenes, although none were significantly different (*p* > 0.05).

### 2.6. Psyllid Attraction

**Figure 6:**
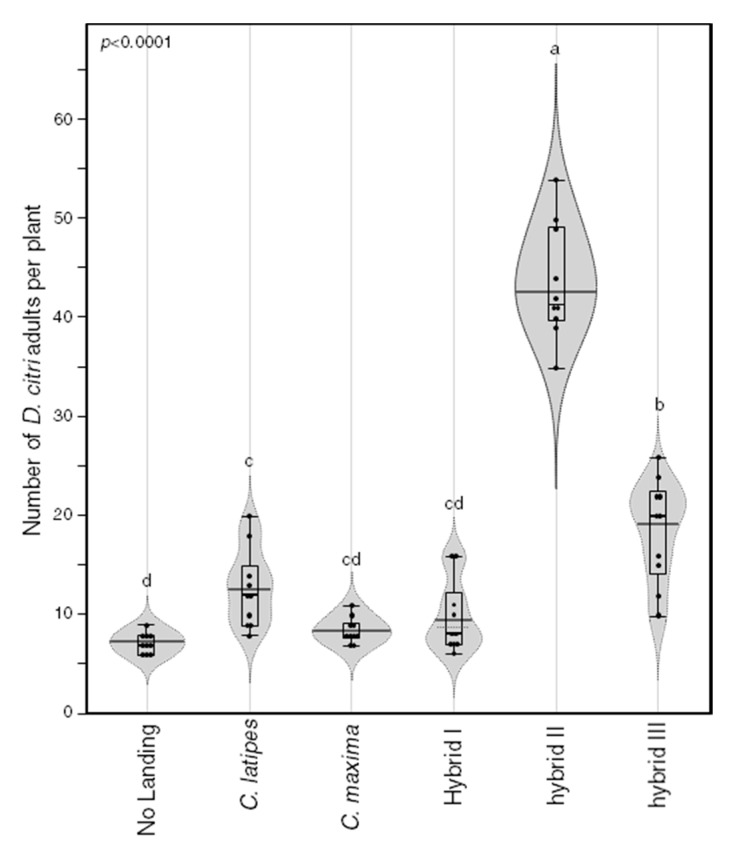
Settling preference of *D. citri* on *C. maxima*, *Citrus latipes* and their corresponding sexual hybrids (Hybrid I, Hybrid II and Hybrid III). Horizontal thick black and gray lines indicate the medians and means (*n = 10*), respectively. Black dots represent individual data points. Boxes show the interquartile ranges including 25–75% of the values, whiskers reflect the highest and the lowest value of data. Gray violin polygons represent the estimated density of the data. Different letters indicate statistically significant differences among treatments using the Tukey–Kramer honest significant difference test (Tukey HSD; *p* < 0.05).

To examine the effect of released volatiles on the attraction of *D. citri*, we conducted the preference assay using the parents and their offspring. The preference assay was repeated ten times in ten different designs to exclude the effect of orientation of host plants (Figure 6). Hybrid II was the most attractive to *D. citri* (40% landing) followed by hybrid III (22%) and *C. latipes* (14%; Figure 6). Pummelo and hybrid I were the least attractive to *D. citri* and the number of psyllids attracted to each of these two hosts was not significantly different from the number of psyllids that did not make any choice (Figure 6).

## 3. Discussion

The three new hybrids generated from the crossing of C2-5-12 Pummelo (*Citrus maxima* (L.) Osbeck) × *C. latipes* were confirmed using morphological phenotype and SSR. The hybrids’ leaf shape was large and showed a medium wing, which was an intermediate between that of their parents. In addition, the new hybrids showed compact and upright tree architecture as their pollen parent (*C. latipes*). The shape of the hybrids’ leaves indicated that they were true sexual hybrids from their parents. The SSR showed that several alleles were present in the hybrids that matched those of the parents, indicating that the offspring were related to their parents. Furthermore, for each progeny the SSR showed several allele combinations that would only be possible in a hybrid. Taken together, these findings indicated a successful crossing between Pummelo and *C. latipes*.

Although there were some differences in the intensity of polar metabolites between the new hybrids and their parents, the GC-MS analysis did not reveal unique marker compounds that can differentiate between the selected species. Consequently, the secondary metabolites of the selected hybrids and their parents should be further investigated using a high-performance liquid chromatography-mass spectrometry (HPLC-MS).

Our results showed that the volatile profile of the Pummelo parent (*C. maxima*) was different from *C. latipes*. In general, *C. latipes* (47 VOCs) contained higher levels of VOCs than Pummelo (28 VOCs). In addition, the levels of most of the detected volatiles in Pummelo were lower than those detected in *C. latipes*. In agreement with this result, *C. latipes* showed the highest level of total aldehydes and total monoterpenes, and was the second highest variety in total volatiles and the third in total sesquiterpenes, among the citrus accessions we tested [11]. *C. latipes* was the highest among fourteen cultivars in neral, undecanal, β-phellandrene, *δ*-elemene, linalool and *δ*-terpinolene [11].

The GC-MS analysis of the hexane extract showed the hybrids were different from their parents. In general, the new hybrids contained higher amounts of VOCs compared to their parents and were more similar to *C. latipes* than Pummelo. For example, the synthesis of several compounds such as neral, geranial, thymol, geranyl acetate and β-myrcene was enhanced in the new hybrids compared to their parents. In addition, hybrid II contained higher amounts of volatiles compared with the other hybrids, and it was the highest in total monoterpenes and second in sesquiterpenes. Interestingly, several compounds were absent in Pummelo but were detected in one or more of the new hybrids, suggesting that they were inherited from *C. latipes*.

Citrus leaf volatiles have been extensively used to study the variation and similarities between citrus species, donor parents and their hybrids, and between hybrids themselves. Gancel et al. (2005) studied the leaf volatile compounds of six citrus somatic allotetraploid hybrids resulting from various combinations using solvent extraction and GC-MS. The chemical analysis showed that hybrids produced from the citrus parent exhibited the same relative contents in terpene hydrocarbons and oxygenated compounds as the acid citrus, while the (grapefruit × orange) hybrid behaved similarly to its two parents [18]. Some of the compounds that were detected in the new hybrids were only detected in one of their parents. On the other hand, some compounds were detected in both parents but were absent in the new hybrids [18]. In agreement with Gancel et al., (2005), our results showed that most compounds detected in *C. latipes* were detected in its hybrids except for three compounds. For example, β-phellandrene was absent in Pummelo, but was detected at a high level in hybrid II, indicating that it was inherited from *C. latipes*. In addition, all compounds that were missing in one or more of the new hybrids were missing at least in one of their parents. These results suggest that the metabolomic analysis could shed insights about the new hybrids and their biochemical relationship to their parents.

The levels of most volatiles in the hexane extract of hybrid II were higher than those detected in its parents and other two hybrids. In the same manner, the levels of most released volatiles from hybrid II were higher than those released from its parents. The preference assay showed that hybrid II was more attractive to *D. citri* than its parents and the other two hybrids. These results indicated that the volatile compounds released from hybrid II were attractive to *D. citri*. Volatiles released from the host plants help insect herbivores to find suitable hosts for feeding and reproduction [19]. Previous studies showed that *D. citri* uses both visual and olfactory cues to find its host plants [8]. Additionally, adult *D. citri* was attracted to mixtures of citrus volatiles [6,20]. Coutinho-Abreu et al. (2014) identified a three-odor blend (β-myrcene, ethyl butyrate and *p*-cymene) out of 61 individually tested odorants with strong antennal sensilla responses of *D. citri*: [20]. In our current work, *β*-myrcene was detected in significant abundance in *C. latipes*, Pummelo and hybrid II leaves via SPME, while *p*-cymene was present in Pummelo and hybrid II leaves but not detected in *C. latipes*. Likewise, *β*-myrcene was detected in significant abundance in the hexane extract of the three new hybrids and their parents. On the other hand, *p*-cymene was present in low abundance in the hexane extracts of *C. latipes* and hybrid II only. Coutinho-Abreu et al. demonstrated that even very low concentrations of some odors had strong stimulatory effects and that the blend of volatiles was more excitatory to olfactory neurons than individual compounds [20]. In bean aphids, Webster et al. (2010) found that the aphids responded more positively to VOC blends mimicking *Vicia faba* volatiles than to the individual components, [21]. A dose-response was observed for many volatiles, with higher doses being repellent, perhaps because higher levels would typically be indicative of herbivory or other mechanical damage to plants [21]. It is widely recognized that single olfactory cues would be unusual, as plants ubiquitously emit VOCs as complex mixtures [22,23], and insects may perceive single odors as non-host plants [21].

In agreement with the hexane extract results, the percentage of monoterpenes released (using SPME) from hybrid II (84%) was higher than that released from *C. latipes* (70%) and Pummelo (67%). On the other hand, the percentage of sesquiterpenes released from hybrid II (5%) was lower than those released from *C. latipes* (7%) and Pummelo (16%). The level of γ-cadinene, δ-cadinene, γ-murrolene, trans-β-farnesene and germacrene D released by Pummelo was higher than that released by hybrid II. Previous studies showed that citrus plants emit a mixture of VOCs, however monoterpenes were the most predominant group within this mixture [9,20,24]. The percentages of monoterpenes and sesquiterpenes released by juvenile sweet orange leaves were 70% and 20%, respectively [9], whereas the percentages of monoterpenes and sesquiterpenes released by mature leaves were 93% and 3%, respectively [9]. Patt and Se´tamou (2010) also found that monoterpenes were the major volatiles emitted by young shoots of Rio Red grapefruit, Meyer lemon and orange jasmine [6]. Besides, *D. citri* was attracted to a mixture of monoterpenes and sesquiterpenes in the following proportions: 5:β-ocimene, 1:d-limonene, 3:β-caryophyllene, 1:α-cubebene, 1:linalool and 1:myrcene (*v/v*) [6]. Our result suggested that the high levels of some released citrus volatiles; especially monoterpenes, could make citrus plants more attractive to *D. citri.* In addition, the ratio of emitted monoterpenes to sesquiterpenes could affect the attraction of *D. citri* to its host plant.

Although the level of total volatile content in hybrid I was similar to that of hybrid II, hybrid I was less attractive to *D. citri* than hybrid II. The GC-MS analysis showed that the percentages of monoterpenes (25%) and sesquiterpenes (6%) in hybrid I were lower than hybrid II (34, and 16%, respectively), however, the percentage of aldehydes (58%) in hybrid I was higher than hybrid II (36%). Interestingly, the level of citronellal, which is a well-known mosquito repellent, was high in hybrid I, indicating that it could have a deterrent effect on *D. citri*. In addition, *C. latipes* released more citronellal, geranial and neral than hybrid II, indicating that they could act as repellants to *D. citri*. Recently, we were able to detect 1,8-cineole (eucalyptol), which is also a well-known mosquito repellent, in “Bingo”, indicating that it could have feeding and ovipositional repellency against *D. citri* [14]. Consequently, we believe that future studies should investigate the response of *D. citri* to citronellal and eucalyptol. Our current and previous studies demonstrated that metabolomics could help us understand the biochemical mechanisms underpinning *D. citri* attraction to citrus plants. 

## 4. Material and Methods

### 4.1. Hybridization and Seedling Selection

Crosses were made in the spring of 2013 using Pummelo C2-5-12 (*Citrus maxima*) as the female parent and *C. latipes* as the pollen parent. C2-5-12 is an open-pollinated seedling of the Florida Department of Plant Industry (DPI) Pummelo cultivar “Ling Ping Yau”, originally selected as a parent for citrus rootstock improvement research. *C. latipes* pollen was obtained from the USDA’s National Clonal Germplasm Repository for Citrus at Riverside, CA (CRC 3052). Putative hybrid seeds were sown in the late fall in bins of calcareous soil (pH, 8), inoculated with *Phytophthora nicotianae* and *P. palmivora* cultures obtained from Dr. Jim Graham (CREC). Robust seedlings were selected based on the growth rate, health and color and transferred to 4 × 4 pots in commercial potting soil (Metromix 500, Sun Gro Horticulture, Bellevue, WA, USA). These seedlings were propagated as cuttings in the mistbed. Well-rooted cuttings were used for all subsequent experiments. 

### 4.2. Genomic DNA Extraction

Genomic DNA was isolated from 4-year-old greenhouse grown hybrids of *C. maxima* × *C. latipes*. Fully expanded leaf tissues (100 mg) were frozen in liquid nitrogen and disrupted using a TissueLyser II (Qiagen^®^, Valencia, CA, USA) for quick pulverization. Tissue lysis was performed at 30 Hz for 1 min and total DNA was extracted with GeneJET plant genomic DNA extraction kit (Thermo-Fisher Scientific, Waltham, MA, USA) according to the manufacturer’s protocol. The concentration of DNA was determined using the NanoDrop^®^ ND-1000 spectrophotometer (Thermo Fisher Scientific). The DNA samples were diluted to the concentration of 25 ng·μL^−1^ in distilled water and used for expressed sequence tag-simple sequence repeat (EST-SSR) analysis.

### 4.3. Plant Genotyping Using SSR Markers

EST-SSR analysis was used to verify the genotype of the new seedlings. SSR analysis was performed according to our previous study [25], using Genemarker^®^ v2.6.3 analysis software v2.6.3 (SoftGenetics^®^, State College, PA, USA) to generate allele tables and graphs. To identify zygotic seedlings of *Citrus maxima* × *Citrus latipes*, eight primers, each with two expected alleles were utilized as described in a previous study [25]. Four fluorescently labeled universal M13 primers, using 6FAM, VIC, NED and PET, were synthesized by Applied Analyzer (3130 XL, Applied Biosystems^®^, Foster City, CA, USA). Fluorescently labeled EST-SSR PCR products were fractionated, and chromatographic peaks were analyzed and exported into an Excel file using GeneMarker^®^ analysis software.

### 4.4. Extraction and Analysis of Polar Metabolites

Briefly, leaves collected from each tree (three mature leaves) were pooled together for analysis. Four biological replicates from each of three hybrids and their parent plants were analyzed, for a total of 20 samples. Pooled leaves from each sample were cut into small pieces using a razor blade and 0.1 g of each sample was placed into a 2 mL screw-cap tube with one 5 mm stainless steel ball. Sample tubes were placed into liquid nitrogen for 5 min and then homogenized using a TissueLyser II (Qiagen, CA) for 1 min at 30 Hz. Polar metabolites were extracted using a mixture of methanol: water (1:1, *v/v*) as described in our previous work [13]. Of each extract 180 µL was placed into a silanized GC vial, spiked with 10 µL ribitol (1 µL·mL^−1^ as an internal standard), dried under a gentle nitrogen stream, derivatized using the trimethylsilylation (TMS) procedure, and analyzed by GC-MS conditions as described by [13]. 

### 4.5. Hexane Extraction and Analysis of Citrus Leaf Volatiles 

Citrus leaf volatile compounds were extracted using hexane as described previously [18] with slight modifications. Briefly, three leaves (young, but fully expanded) were collected and pooled together for analysis. Five different biological samples were collected for analysis from each parent tree and the three hybrids. Pooled leaves were prepared for analysis identically as above (0.1 g frozen and homogenized), except that 0.5 mL of hexane was added to extract the volatile components instead of the methanol/water mixture. The tubes were placed on ice and sonicated for 5 min, then placed on a tube rotator for 1 h at 8 °C. Following extraction, the samples were centrifuged for 3 min at 10,000 rpm and 0.2 mL of supernatant was transferred to an amber vial with 350 µL fused inserts (C4000-LV2, National Scientific, Rockwood, TN, USA). Each sample was spiked with *trans*-2-nonenal at a final concentration of 1 µL·mL^−1^ as an internal standard. Aliquots of the hexane extracts (0.5 µL) were injected into the GC-MS using the same conditions described in our previous study [13].

### 4.6. Collection and Analysis of Released Volatiles

Released citrus volatile organic compounds (VOCs) were collected by static headspace solid phase microextraction (SPME) following the design described previously [9] for the collection of VOCs from living citrus plants (in vivo SPME). Briefly, an intact juvenile leaf bundle was inserted into a plastic tube and sealed at the stem end by Parafilm. The SPME fiber (StableFlex^™^ 1 cm triple-coated 50/30 μm carboxen/divinylbenzene/polydimethylsiloxane; #57328-U, Supelco, Bellefonte, PA, USA) was inserted and sealed into the opposite end with parafilm. After 5 min of equilibration, the SPME fiber was exposed to the citrus leaves for 2 h at 27 °C and then was carefully retracted. After the collection of released leaf volatiles, the SPME fiber was inserted into the GC inlet for 5 min at 250 °C for thermal desorption.

### 4.7. Identification and Quantification of Leaf VOCs and Polar Metabolites 

The citrus leaf VOCs and polar metabolites were separated by gas chromatography-mass spectrometry (GC-MS) using the same chromatographic conditions as reported in our previous study [9]. Peaks of interest were identified by comparing their mass spectra to those of authentic standards and by matching them to one of two mass spectral libraries with a score of at least 700 (Wiley Flavor and Fragrances of Synthetic and Natural Compounds or NIST 2011 Mass Spectral Database). Reference compounds were purchased from Sigma-Aldrich (St. Louis, MO, USA) at the highest available purity, and injected into the GC-MS under the same conditions as experimental samples to establish their retention time on the column. Peak areas were integrated using TurboMass software v. 5.4.2 (Perkin Elmer, Waltham, MA, USA), and were normalized to the area of the internal standard, *trans*-2-nonenal for VOCs and ribitol for polar metabolites. Quantification of compounds was based on the peak areas obtained from a dilution series of reference standards at known concentrations. Calibration curves were constructed from the linear regressions obtained by plotting the concentration vs. peak area for each reference compound at each concentration.

### 4.8. Psyllid Attraction

Colonies of *D. citri* were reared on HLB-free curry leaf plants (*Bergera* (*Murraya*) *koenigii*) in a growth room maintained at 28 ± 1 °C and 60% ± 5% relative humidity, and with a 16:8 h (light: dark) photoperiod. Only adult psyllids were collected for host preference experiments.

To evaluate the settling preference of *D. citri*, one plant (1-year old) from each hybrid and parent was placed equidistantly inside a 60 cm × 60 cm × 90 cm insect cage (#1466CV, Bioquip Products, Rancho Dominguez, CA, USA) and 100 *D. citri* adults were released from a collection vial placed in the center of the cage. The order of the plants within the cages was randomized to minimize any positional bias. Psyllids were released in the afternoon and collected the following morning (18 h), allowing settling to occur by both visual and olfactory cues (in the light and dark). The number of *D. citri* settling on each plant variety was recorded. Ten cages were used in this experiment and each cage was considered a single replicate.

### 4.9. Statistical Analysis

Statistical analyses were performed using JMP version 9.0 (SAS Institute Inc., Cary, NC, USA). Data were normally distributed. Each treatment was composed of four biological replicates. An analysis of variance (ANOVA), followed by post hoc pairwise comparisons with the Tukey–Kramer honest significant difference test (Tukey HSD) was used to compare the level of each metabolite in the different species. The score and loading plot for non-volatile compounds was generated using a principal component analysis (PCA). Additionally, a two-way hierarchical cluster analysis (HCA) and heat maps were generated using the means of the data matrices. Distance and linkage were done using the Bray-Curtis similarity measure method [26]. The two-way HCA and heat map of released volatiles from *C. maxima, C. latipes* and their promising sexual hybrid (Hybrid II) were generated using the means of the data matrices based on the Ward’s minimum variance method [27]. The number of insects settling on each variety was analyzed by ANOVA and were compared using Tukey’s honest significant difference (HSD) test. 

## 5. Conclusions

Our results showed that metabolomic analysis could reveal significant insights about the new hybrids and their relationship with each other and their parents. The levels of released monoterpenes from hybrid II, which was attractive to *D. citri*, were higher than those released from its parents. This result suggested that monoterpenes could play an important role in the attraction of *D. citri*. Our results suggested that hybrid II could be used as a windbreak and trap tree for *D. citri* (double duty), once its tolerance to HLB is confirmed. Finally, our current study suggested that metabolomics could help understand the mechanism behind the attraction of *D. citri* to its citrus host plants.

## Figures and Tables

**Table 1:** Simple Sequence Repeat of *C. maxima* and *C. latipes* and their hybrids (*C. maxima* × *C. latipes*) using eight SSR primers.

	EST-SSR Markers ^a^ (Base Pair)															
	*CX6F02B*		*CX6F04G*		*CX6F06Y*		*CX6F10R*		*CX6F17B*		*CX6F18G*		*CX6F29Y*		*CX0035R*	
*C. maxima*	168	168	163	169	157	157	176	176	132	132	160	161	152	152	183	183
*C. latipes*	168	168	157	169	166	166	170	170	126	132	148	148	152	152	183	183
Hybrid I	168	168	169	169	157	166	170	176	126	132	148	160	152	152	183	183
Hybrid II	168	168	169	169	157	166	170	176	126	132	148	161	152	152	183	183
Hybrid III	168	168	169	169	157	166	170	176	126	132	148	160	152	152	183	183

^a^ EST-SSR primers amplified amplicon size from chromatogram. Three of these primers (CX6F02B, CX6F29Y, and CX0035R) revealed no polymorphism and were uninformative.

**Table 2:** Concentration (µg·g^−1^ FW) of different hexane-extracted volatile organic compounds detected in leaves of C2-5-12 Pummelo (*Citrus maxima*), *C. latipes* and three corresponding sexual hybrids using gas chromatography-mass spectrometry (*n* = 5) ^z^.

Peak No.	VOC ^y^	RT	*Citrus maxima*	*Citrus latipes*	Hybrid I	Hybrid II	Hybrid III	*p*-Value ^x^
1	*α*-Thujene ^v^	7.78	ND	ND	0.01 ± 0.01 ^b^	0.22 ± 0.20 ^a^	0.01 ± 0.01 ^b^	**0.0091**
2	*α*-Pinene ^v^	7.95	0.02 ± 0.01 ^c^	1.92 ± 0.65 ^ab^	0.47 ± 0.33 ^bc^	2.65 ± 1.30 ^a^	0.25 ± 0.22 ^c^	**0.0002**
3	Sabinene ^v^	8.90	0.07 ± 0.04 ^b^	1.15 ± 0.45 ^b^	0.80 ± 0.50 ^b^	3.05 ± 1.05 ^a^	0.69 ± 0.31 ^b^	**<0.0001**
4	*β*-Pinene ^v^	9.00	0.51 ± 0.25 ^b^	0.05 ± 0.06 ^b^	4.53 ± 2.38 ^b^	11.84 ± 4.50 ^a^	4.08 ± 1.29 ^b^	**<0.0001**
5	*β*-Myrcene ^u^	9.25	0.04 ± 0.02 ^c^	8.81 ± 3.30 ^c^	46.38 ± 17.31 ^a^	13.70 ± 2.53 ^bc^	31.80 ± 9.98 ^ab^	**<0.0001**
6	*α*-Phellandrene ^s^	9.66	0.04 ± 0.01 ^c^	4.26 ± 1.30 ^a^	0.14 ± 0.10 ^c^	1.98 ± 0.24 ^b^	0.13 ± 0.09 ^c^	**<0.0001**
7	*γ*-Carene ^u^	9.71	ND	0.15 ± 0.15 ^ns^	0.04 ± 0.03 ^ns^	0.03 ± 0.01 ^ns^	0.09 ± 0.09 ^ns^	0.1076
8	*δ*-Carene ^u^	9.90	0.09 ± 0.16 ^b^	1.04 ± 0.39 ^a^	0.02 ± 0.01 ^b^	1.25 ± 0.26 ^a^	0.02 ± 0.02 ^b^	**<0.0001**
9	*ρ*-Cymene ^w,t^	10.05	ND	0.03 ± 0.02 ^ns^	ND	0.09 ± 0.09 ^ns^	ND	0.3062
10	d-Limonene ^s^	10.20	0.14 ± 0.07 ^c^	19.30 ± 10.79 ^b^	30.70 ± 6.61 ^ab^	35.59 ± 6.22 ^a^	24.21 ± 4.76 ^ab^	**<0.0001**
11	*β*-Phellandrene ^w,s^	10.30	ND	17.34 ± 4.67 ^a^	ND	8.14 ± 2.09 ^b^	ND	**0.0214**
12	Z-*β*-Ocimene ^s^	10.60	0.12 ± 0.05 ^c^	1.79 ± 0.65 ^b^	3.11 ± 0.70 ^ab^	3.73 ± 0.90 ^a^	2.88 ± 0.55 ^ab^	**<0.0001**
13	*γ*-Terpinene ^s^	10.70	0.37 ± 0.08 ^b^	0.53 ± 0.10 ^b^	0.69 ± 0.18 ^b^	3.58 ± 1.13 ^a^	0.78 ± 0.10 ^b^	**<0.0001**
14	Unknown terpene alcohol 1 ^r^	11.25	0.20 ± 0.17 ^b^	0.54 ± 0.31 ^ab^	1.07 ± 0.60 ^a^	0.96 ± 0.23 ^ab^	1.16 ± 0.40 ^a^	**0.0117**
15	Unknown terpene alcohol 2 ^r^	11.35	ND	0.11 ± 0.03 ^ab^	0.17 ± 0.05 ^a^	0.16 ± 0.02 ^ab^	0.18 ± 0.05 ^a^	**0.0132**
16	*α*-Terpinolene ^s^	11.50	0.01 ± 0.01 ^b^	0.06 ± 0.06 ^b^	0.05 ± 0.07 ^b^	0.27 ± 0.05 ^a^	0.06 ± 0.04 ^b^	**<0.0001**
17	Linalool ^r^	11.80	0.55 ± 0.12 ^b^	14.02 ± 3.53 ^a^	4.34 ± 0.50 ^b^	10.95 ± 1.81 ^a^	4.30 ± 0.69 ^b^	**<0.0001**
18	Citronellal ^q^	12.90	ND	42.73 ± 8.32 ^ab^	53.40 ± 12.39 ^a^	28.14 ± 6.97 ^bc^	19.35 ± 0.87 ^c^	**<0.0001**
19	Indole ^w,r^	13.41	ND	0.11 ± 0.16 ^ns^	0.02 ± 0.03 ^ns^	ND	ND	0.3786
20	Terpinen-4-ol ^r^	13.51	ND	0.02 ± 0.02 ^b^	0.30 ± 0.21 ^a^	0.22 ± 0.07 ^ab^	0.33 ± 0.11 ^a^	**0.0431**
21	*α*-Terpineol ^r^	13.86	ND	0.53 ± 0.21 ^b^	0.09 ± 0.02 ^c^	0.81 ± 0.10 ^a^	ND	**<0.0001**
22	Piperitol ^w,r^	14.16	ND	0.56 ± 0.28 ^b^	ND	1.31 ± 0.51 ^a^	ND	**0.0350**
23	Nerol ^r^	14.45	ND	0.55 ± 0.16 ^a^	0.15 ± 0.17 ^b^	0.12 ± 0.01 ^b^	0.14 ± 0.06 ^b^	**<0.0001**
24	Neral ^p^	14.75	0.14 ± 0.05 ^b^	1.17 ± 1.73 ^b^	64.99 ± 29.56 ^a^	56.58 ± 15.11 ^a^	67.42 ± 6.82 ^a^	**<0.0001**
25	Geraniol ^r^	14.95	ND	0.27 ± 0.09 ^b^	1.25 ± 0.30 ^a^	1.17 ± 0.12 ^a^	0.95 ± 0.19 ^a^	**<0.0001**
26	Geranial ^p^	15.36	0.60 ± 0.55 ^b^	2.25 ± 1.62 ^b^	94.98 ± 44.93 ^a^	83.56 ± 22.25 ^a^	95.57 ± 41.18 ^a^	**0.0002**
27	Thymol ^r^	15.75	0.28 ± 0.07 ^c^	0.40 ± 0.07 ^bc^	0.49 ± 0.08 ^ab^	0.50 ± 0.03 ^ab^	0.53 ± 0.09 ^a^	**0.0007**
28	*δ*-Elemene ^o^	16.77	ND	10.45 ± 3.08 ^b^	0.37 ± 0.14 ^c^	15.09 ± 2.46 ^a^	0.20 ± 0.09 ^c^	**<0.0001**
29	Neryl acetate ^p^	17.10	0.23 ± 0.09 ^b^	1.54 ± 0.40 ^a^	0.57 ± 0.20 ^b^	0.64 ± 0.17 ^b^	0.28 ± 0.03 ^b^	**<0.0001**
30	Geranyl acetate ^p^	17.45	0.01 ± 0.01 ^d^	2.90 ± 0.84 ^cd^	12.48 ± 1.66 ^ab^	14.62 ± 4.61 ^a^	8.97 ± 1.67 ^c^	**<0.0001**
31	*β*-Elemene ^o^	17.70	0.02 ± 0.01 ^c^	0.08 ± 0.04 ^b^	0.10 ± 0.01 ^ab^	0.15 ± 0.02 ^a^	0.07 ± 0.04 ^c^	**<0.0001**
32	Sesquithujene ^o^	17.90	0.03 ± 0.01 ^ns^	0.04 ± 0.00 ^ns^	0.05 ± 0.01 ^ns^	0.05 ± 0.02 ^ns^	0.04 ± 0.02 ^ns^	0.1753
33	Anthranilate methyl ester ^w,p^	18.05	ND	0.31 ± 0.07 ^ns^	ND	ND	0.34 ± 0.22 ^ns^	0.5573
34	Unknown Ketone/Eucarvone ^p^	18.20	0.02 ± 0.00 ^b^	0.02 ± 0.01 ^b^	0.15 ± 0.07 ^ab^	0.24 ± 0.05 ^a^	0.20 ± 0.18 ^ab^	**0.0071**
35	*trans*-β-Caryophyllene ^o^	18.33	0.09 ± 0.04 ^c^	3.14 ± 0.55 ^a^	3.54 ± 0.61 ^a^	2.59 ± 0.59 ^ab^	2.12 ± 0.26 ^b^	**<0.0001**
36	Aromadendrene ^o^	18.50	ND	0.12 ± 0.04 ^b^	0.05 ± 0.01 ^bc^	0.32 ± 0.08 ^a^	0.02 ± 0.02 ^c^	**<0.0001**
37	*trans*-β-Farnesene ^o^	18.88	ND	0.25 ± 0.06 ^a^	0.22 ± 0.04 ^a^	0.22 ± 0.02 ^a^	0.11 ± 0.06 ^b^	**<0.0001**
38	*α*-Humulene ^o^	19.03	ND	0.34 ± 0.09 ^a^	0.32 ± 0.19 ^a^	0.32 ± 0.06 ^a^	0.16 ± 0.09 ^b^	**0.0013**
39	Valencene ^o^	19.75	ND	3.92 ± 0.77 ^bc^	4.60 ± 1.13 ^ab^	6.21 ± 1.67 ^a^	2.21 ± 0.37 ^c^	**<0.0001**
40	Benzoic acid,4-hydroxy ^p^	20.11	5.49 ± 2.17 ^b^	7.95 ± 0.69 ^ab^	8.05 ± 0.62 ^a^	8.23 ± 0.52 ^a^	8.23 ± 1.02 ^a^	**0.0201**
41	Nerolidol ^n^	20.25	ND	0.63 ± 0.08 ^a^	0.44 ± 0.10 ^b^	0.38 ± 0.09 ^bc^	0.24 ± 0.07 ^c^	**<0.0001**
42	Sesquiterpene Alcohol ^n^	21.12	0.03 ± 0.01 ^c^	0.68 ± 0.13 ^ab^	0.90 ± 0.18 ^a^	1.02 ± 0.15 ^a^	0.47 ± 0.24 ^b^	**<0.0001**
43	*α*-Farnesol ^n^	22.75	0.11 ± 0.03 ^ns^	0.12 ± 0.02 ^ns^	0.15 ± 0.03 ^ns^	0.15 ± 0.04 ^ns^	0.14 ± 0.07 ^ns^	0.5531
44	*β*-Farnesol ^n^	22.85	0.12 ± 0.03 ^ns^	0.13 ± 0.03 ^ns^	0.18 ± 0.04 ^ns^	0.18 ± 0.05 ^ns^	0.16 ± 0.11 ^ns^	0.5697
45	Psoralen ^n^	25.00	0.01 ± 0.00 ^b^	0.03 ± 0.03 ^b^	0.16 ± 0.02 ^a^	0.13 ± 0.09 ^ab^	0.13 ± 0.08 ^ab^	**0.0060**
46	Methoxalen ^n^	28.20	ND	0.39 ± 0.23 ^b^	0.07 ± 0.03 ^c^	0.44 ± 0.13 ^a^	0.11 ± 0.12 ^bc^	**0.0005**
47	Phytol ^n^	28.95	0.04 ± 0.04 ^ns^	0.04 ± 0.01 ^ns^	0.76 ± 1.37 ^ns^	0.07 ± 0.04 ^ns^	1.14 ± 1.54 ^ns^	0.3385
**Total VOCs**			**9.37 ± 4.10 ^c^**	**152.78 ± 46.31 ^b^**	**341.35 ± 123.53 ^a^**	**321.67 ± 78.64 ^a^**	**280.29 ± 74.23 ^a^**	**<0.0001**

^z^ Values represent means ± SD (*n* = 5). ^y^ Quantification of leaf volatile organic compounds was based on calibration curves obtained from standards of known concentration injected into the GC-MS (Perkin Elmer Elite-5 ms, 30 m × 0.25 mm × 0.25 µm) under the same chromatographic conditions as the samples. ND: compound under the limit of detection. ^x^ *p*-values are bolded if less than 0.05. Different letters (a,b,c) next to values indicate statistically significant differences among the studied varieties (*p* < 0.05), while ns signifies no significant differences among them using the Tukey–Kramer honest significant different test (Tukey HSD). ^w^ Different letters indicate statistically significant differences among the studied varieties (*p* < 0.05), while cells without letters or with the same letter signify no significant differences among them using Student’s *t*-test. ^v^ VOC quantified using sabinine authentic reference standard. ^u^ VOC quantified using *β*-myrcene authentic reference standard. ^t^ VOC quantified using *p*-cymene authentic reference standard. ^s^ VOC quantified using d-limonene authentic reference standard. ^r^ VOC quantified using linalool authentic reference standard. ^q^ VOC quantified using citronellal authentic reference standard. ^p^ VOC quantified using neral authentic reference standard. ^o^ VOC quantified using caryophyllene authentic reference standard. ^n^ VOC quantified using nerolidol authentic reference standard.

## Supplementary Materials

The following are available online at https://www.mdpi.com/2218-1989/10/12/477/s1, Table S1: Concentrations (μg·g^−1^ FW) of the major polar metabolites detected in leaves of C2-5-12 Pummelo (*Citrus maxima*), *C. latipes*, and three corresponding sexual hybrids after derivatization with TMS and using gas chromatography-mass spectrometry.
