# Supplementary material for: Metabolic Profiling of Hybrids Generated from Pummelo and Citrus latipes in Relation to Their Attraction to Diaphorina citri, the Vector of Huanglongbing
Source: Metabolites. 2020 Nov 24;10(12):477. doi: 10.3390/metabo10120477 (PMC7760127; doi:10.3390/metabo10120477)
Supplement: Supplementary file 1 [file metabolites-10-00477-s001.pdf]

Table S1. Concentrations (μg.g^{-1} FW) of the major polar metabolites detected in leaves of C2-5-12 Pummelo (*Citrus maxima*), *C. latipes*, and three corresponding sexual hybrids after derivatization with TMS and using gas chromatography-mass spectrometry (*n*=4) [z].

Peak No.	TMS Metabolite [y]	*Citrus maxima*	*Citrus latipes*	Hybrid 1	Hybrid II	Hybrid III	*p*-value [x]
1	Pyruvic acid	2.19±1.65	1.91±1.52	2.43±1.65	2.23±1.73	2.13±1.67	0.1304
2	Lactic acid [w]	4.10±1.99 [ab]	4.87±2.89 [a]	2.45±1.86 [b]	2.43±1.85 [b]	2.84±1.84 [b]	**0.0043**
3	L-Alanine [w]	6.65±2.26	8.56±3.29	7.55±3.44	10.82±3.97	8.88±2.36	0.2384
4	N-methyl proline	4.71±1.55	8.22±3.79	4.97±1.37	8.01±3.05	5.47±1.66	0.0614
5	L-Valine [w]	2.70±1.39	3.68±1.37	3.47±1.64	3.58±1.52	3.17±1.93	0.3536
6	Benzoic acid [w]	7.96±2.45	6.78±2.06	7.32±3.53	8.85±3.16	7.72±2.40	0.7105
7	Phosphoric acid [w]	42.30±14.83 [ab]	58.12±17.97 [a]	24.46±10.89 [b]	25.96±12.03 [b]	34.28±8.74 [ab]	**0.0205**
8	Glycerol [w]	18.30±3.94	19.42±2.28	19.25±4.97	23.76±3.98	20.47±3.95	0.3037
9	L-Proline [w]	100.03±30.94 [ab]	159.74±44.55 [a]	71.98±12.82 [b]	102.99±14.59 [ab]	85.72±23.29 [b]	**0.0127**
10	Propanoic acid, 1-oxo	7.94±4.73	7.43±3.07	5.91±2.53	7.45±3.40	6.42±3.02	0.7391
11	L-Serine [w]	50.87±23.77	55.78±15.54	41.23±5.40	51.74±9.40	42.97±10.90	0.6157
12	L-Threonine [w]	120.42±40.88 [a]	36.01±13.94 [b]	67.18±19.97 [ab]	69.47±23.70 [ab]	89.90±14.80 [ab]	**0.0067**
13	Malic acid [w]	161.81±54.91 [a]	48.36±18.71 [b]	90.26±26.82 [ab]	93.33±31.83 [ab]	120.79±19.87 [ab]	**0.0067**
14	L-Aspartic acid [w]	7.96±3.12 [b]	32.45±12.69 [a]	4.60±2.81 [b]	7.05±4.44 [b]	8.29±4.11 [b]	**<0.0001**
15	γ-Aminobutyric acid [w]	67.82±12.58	83.91±20.93	76.95±17.40	82.67±11.12	83.26±23.40	0.7724
16	Arabinofuranose	5.93±2.02	6.53±2.29	4.31±2.17	6.34±2.04	4.47±1.54	0.0665
17	Threonic acid [w]	157.79±37.37 [a]	25.39±13.11 [c]	76.56±34.92 [b]	48.00±25.68 [bc]	70.00±33.50 [bc]	**<0.0001**
18, 19	Xylose [w]	31.09±13.08	32.27±14.51	19.92±9.47	27.40±11.08	23.89±10.45	0.6356

Peak No.	TMS Metabolite [y]	*Citrus maxima*	*Citrus latipes*	Hybrid 1	Hybrid II	Hybrid III	*p*-value [x]
20	L-Asparagine [w]	12.38±3.73	11.80±6.84	7.50±4.71	8.76±3.46	8.65±5.03	0.2495
21	Xylitol [w]	5.63±2.59	11.30±2.79	10.28±4.07	9.81±4.01	9.15±2.72	0.1425
22	Ribonic acid	88.45±20.40 [a]	37.26±12.22 [b]	53.58±18.13 [ab]	71.62±20.66 [ab]	46.34±10.23 [b]	**0.0051**
23	Shikimic acid	100.37±6.95 [a]	33.23±12.15 [c]	43.02±17.49 [bc]	69.94±21.32 [ab]	56.22±20.05 [bc]	**0.0004**
24	Citric acid [w]	332.30±94.74 [b]	592.90±154.40 [a]	318.40±55.03 [b]	386.93±58.18 [ab]	382.70±110.12 [ab]	**0.0271**
25	Unknown sugar 1	54.46±15.96	54.03±10.42	55.02±17.37	59.04±4.56	57.00±13.06	0.9849
26	Quinic acid [w]	593.01±185.00 [a]	115.36±43.02 [b]	416.96±173.37 [ab]	224.81±125.74 [b]	212.45±69.26 [b]	**0.0030**
27, 28	Fructose [w]	52.66±19.71	66.51±15.82	56.93±14.18	50.58±12.77	77.97±3.59	0.1248
29, 31	Glucose [w]	92.88±32.10	90.03±15.93	125.67±53.53	93.53±11.40	143.29±66.20	0.4185
30, 32	Mannose [w]	33.15±6.54	31.33±7.65	32.84±3.82	41.22±7.46	32.12±11.17	0.4154
33	Galactose [w]	23.52±12.47	19.82±5.90	29.90±6.07	13.47±5.29	40.71±23.51	0.1095
34	*chiro*-Inositol [w]	2.64±1.83 [c]	20.22±4.65 [a]	18.19±6.49 [ab]	21.83±3.43 [a]	12.55±3.83 [b]	**<0.0001**
35	Gluconic acid [w]	10.21±1.71 [a]	6.59±1.82 [b]	5.86±2.15 [b]	5.09±2.40 [b]	6.55±1.29 [b]	**0.0008**
36	Saccharic acid [w]	6.05±2.89 [b]	22.29±5.46 [a]	6.82±1.22 [b]	8.82±2.72 [b]	10.19±2.43 [b]	**<0.0001**
37	*scyllo*-Inositol [w]	37.47±6.76 [a]	17.50±4.69 [c]	24.67±6.53 [bc]	33.59±5.07 [ab]	28.27±6.55 [abc]	**0.0006**
38	Galactaric acid [w]	1.21±0.88 [b]	2.70±0.25 [a]	1.13±0.81 [b]	1.18±0.85 [b]	1.27±0.84 [b]	**0.0490**
39	Palmitic acid [w]	1.73±0.85	1.40±0.98	1.67±1.05	1.99±1.07	1.61±1.19	0.1865
40	*myo*-Inositol [w]	17.63±5.54	9.85±3.20	16.04±6.10	12.89±4.82	15.99±4.97	0.1053
41	Arabino-hexaric acid	60.60±8.03	46.35±4.52	52.86±10.16	50.42±11.33	59.38±13.10	0.1883
43	Stearic acid [w]	1.92±1.36	2.07±1.10	2.15±0.91	2.15±1.29	2.13±1.57	0.9793
44	Glyceryl-glycoside	6.46±1.64	5.78±1.75	6.34±2.16	6.97±1.63	7.63±2.16	0.3639

Peak No.	TMS Metabolite [y]	*Citrus maxima*	*Citrus latipes*	Hybrid 1	Hybrid II	Hybrid III	*p*-value [x]
45	Sucrose [w]	172.55±35.04 [b]	461.72±106.29 [a]	420.13±57.16 [a]	416.76±58.51 [a]	470.09±76.92 [a]	**0.0004**

[z] Values represent means±SD (*n=4*). [y] Quantification of leaf metabolites was based on calibration curves obtained from standards of known concentration injected into the GC-MS (Perkin Elmer Elite-5ms, 30 m × 0.25 mm × 0.25 µm) under the same chromatographic conditions as the samples. [x] *p*-values are bolded if less than 0.05. Different letters indicate statistically significant differences among the studied varieties ($p < 0.05$), while cells without letters or with the same letter signify no significant differences among them using Tukey-Kramer honestly significant different test (Tukey HSD).[w] Metabolites have been confirmed using authentic reference standard.
